# Small Bowel Malignancies in Patients Undergoing Capsule Endoscopy for Iron Deficiency Anemia

**DOI:** 10.3390/diagnostics12010091

**Published:** 2021-12-31

**Authors:** Su Hwan Kim, Ji Won Kim

**Affiliations:** Department of Internal Medicine, Seoul Metropolitan Government Seoul National University Boramae Medical Center, Seoul National University College of Medicine, 20 Boramae-ro 5-gil, Dongjak-gu, Seoul 07061, Korea; schwann@naver.com

**Keywords:** capsule endoscopy, small bowel malignancy, iron deficiency anemia

## Abstract

Small bowel malignancies are rare and usually asymptomatic or symptoms are nonspecific. Therefore, small bowel tumors are difficult to diagnose. In patients with iron deficiency anemia (IDA) who have negative bidirectional endoscopy results, the small bowel may be considered the source of bleeding. However, in asymptomatic IDA patients with negative bidirectional endoscopy results, evidence supporting the routine use of capsule endoscopy (CE) is insufficient. CE can be considered in selected patients with recurrent or persistent IDA. The frequency of small bowel malignancies is low in patients undergoing CE for IDA, but the usefulness of CE for the diagnosis of small bowel malignancies in younger age groups with IDA has been reported. For patients with risk factors for small bowel malignancy, investigation of the small bowel should be considered. Efforts should be made to prevent adverse events, such as capsule retention or capsule aspiration, through meticulous history taking and endoscopic capsule delivery as necessary.

## 1. Introduction

Obscure gastrointestinal bleeding (OGIB) is defined as bleeding from the gastrointestinal (GI) tract without an obvious etiology after negative bidirectional endoscopy (upper GI endoscopy and colonoscopy) [1,2]. OGIB is subdivided into overt OGIB and occult OGIB—iron deficiency anemia (IDA) and/or positive fecal occult blood test [3]. In patients presenting with overt OGIB, capsule endoscopy (CE) should be performed to evaluate the small bowel [4]. In patients with occult blood in the stool but without anemia, advanced examination beyond bidirectional endoscopy is not recommended [5]. In the 2015 ESGE guidelines, the fecal occult blood test was not recommended as a screening tool to decide whether to perform small bowel CE [6]. In patients with IDA, bidirectional endoscopy to evaluate the GI tract should generally be the first examination [7]. If the bidirectional endoscopy results are negative, then the small bowel can be considered the source of bleeding. In such cases, the use of CE is common in clinical practice [8]. Known causes of small bowel bleeding include angioectasias, tumors, inflammatory bowel diseases, and drug-induced injuries [1]. Small bowel malignancies are rare in patients with IDA; however, it is critically serious. In this study, we reviewed the role and diagnostic yield of capsule endoscopy in patients with IDA. In addition, we investigated the frequency of small bowel malignancies detected through CE in patients with IDA, and briefly reviewed the adverse events associated with CE.

## 2. Small Bowel Malignancies

**Figure 1:**
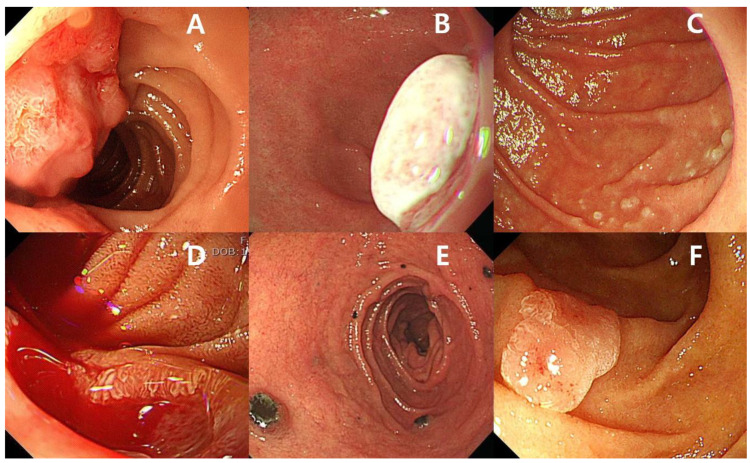
(**A**) Adenocarcinoma of the small bowel. (**B**) Neuroendocrine tumor of the small bowel. (**C**) Follicular lymphoma of the small bowel. (**D**) Gastrointestinal stromal tumor of the small bowel with active bleeding. (**E**) Secondary small bowel malignancy originating from malignant melanoma. (**F**) Adenoma of the small bowel.

The small bowel is very long (6–7 m) and accounts for 75% of the length of the GI tract. Small bowel malignancies are rare, constituting only 1–3% of all GI malignancies [9]. Patients with small bowel tumors are usually asymptomatic or have only nonspecific symptoms, such as GI bleeding, anemia, abdominal pain, vomiting, and weight loss [10,11]. Previously, the diagnosis of small bowel malignancies was difficult because of the length of the small bowel and the limitations of the diagnostic tools [12]. The advent of CE and device-assisted enteroscopy (DAE) has revolutionized the diagnosis and management of small bowel diseases [2]. Small bowel malignancies are subdivided into primary and secondary based on origin. The most frequent types of primary small bowel malignancies are adenocarcinoma (Figure 1A), neuroendocrine tumors (Figure 1B), lymphoma (Figure 1C), and gastrointestinal stromal tumor (GIST) (Figure 1D) [12,13]. Secondary small bowel malignancies can develop by direct invasion or distant metastasis. Frequently, secondary small bowel malignancies originate from malignant melanoma (Figure 1E), as well as lung, breast, and colorectal cancers [14]. Known risk factors associated with primary small bowel cancers are Peutz–Jeghers syndrome (PJS), familial adenomatous polyposis (FAP), Lynch syndrome, Crohn’s disease, and celiac disease. Importantly, adenoma (Figure 1F) of the small bowel is a known precursor of small bowel adenocarcinoma [15,16,17]. Duodenal adenomas are also associated with FAP and Lynch syndrome [18]. The adenoma–carcinoma sequence in the small intestine was described, and the risk of progression to malignant neoplasm was similar to that in the colorectum [19]. The presence of duodenal adenomas, especially in young patients, suggests the possibility of hereditary polyposis syndrome, and indicates the need for colonoscopy to exclude colorectal neoplasms [19,20]. Some studies have reported a relationship between sporadic duodenal adenomas and colorectal adenomas [21,22,23]. An association between an increased risk of small bowel cancer and environmental factors, including alcohol consumption, smoking, red meat, and intake of sugary drinks, was reported, although the evidence is insufficient owing to the rarity of small bowel malignancies [17].

## 3. Gastrointestinal Tract Evaluation of Patients with Iron Deficiency Anemia

IDA is the main cause of anemia and is a main indication for referral to gastroenterologists [24]. Known causes of IDA include inadequate dietary intake, poor absorption, and chronic blood loss [7]. Identification of GI malignancy is a critical concern in patients with IDA. Risk predictors of GI malignancy, including age, sex, hemoglobin level, and mean corpuscular volume (MCV), have been identified in previous studies [25,26]. About 5–12% of healthy premenopausal women have IDA, which is attributed to inadequate dietary intake, menstrual blood loss, pregnancy, and breastfeeding [27,28]. In premenopausal women, the prevalence of GI malignancy is low and bidirectional endoscopy is not generally recommended [29]. However, premenopausal women sometimes develop GI tract cancers or benign bleeding lesions such as peptic ulcers. Therefore, GI investigation of premenopausal women with IDA is justified, particularly in patients with recurrent IDA, symptoms, or a family history of GI malignancy [7,29]. In addition, studies have reported a high frequency of upper GI and colorectal cancers in premenopausal women [30,31].

IDA is rare in young men, but the diagnostic yield of GI tract pathology in endoscopic examination is significantly higher in men than in women of the same age. Thus, GI evaluation at the same level as for the elderly is recommended for young men [25,32]. Men and postmenopausal women have a higher prevalence of upper GI and colorectal malignancies than premenopausal women; thus, bidirectional endoscopy is recommended [7,29]. Some studies have reported high frequencies of upper GI and colorectal cancers in men and postmenopausal women [33,34]. Despite the possibility of selection bias in studies that have reported high cancer rates, considering the minimal risk of endoscopic examination, the benefit of diagnosing malignant lesions outweighs the risk [7].

Anemia is a common finding in the elderly population. The causes of anemia vary. In approximately half of the elderly with anemia, IDA is the cause [29]. Anemia in the elderly has been shown to result in reduced physical performance, muscle strength, and cognitive function, which can result in falls, fractures, hospitalization, depression, and mortality [35,36,37]. In elderly patients with IDA, bidirectional endoscopy to evaluate the GI tract should be considered because of the possibility of clinically significant peptic ulcers or GI malignancy. However, risks and benefits should be considered before endoscopic examination in the elderly, particularly in those with significant comorbidity, frailty, and reduced life expectancy [37]. Complications, risks, therapeutic options, and the benefits of examinations need to be discussed with each elderly patient and their family members before invasive investigation. Bowel preparation and colonoscopy are often burdensome in elderly patients. Therefore, if there is no clear therapeutic benefit, careful consideration is required before a colonoscopy is performed [37].

## 4. Role of Capsule Endoscopy in Patients with Iron Deficiency Anemia

In patients with unexplained IDA, CE is recommended as the first diagnostic test to evaluate the small bowel [38]. Before the advent of CE and DAE, small bowel follow-through, abdominopelvic CT scan, push enteroscopy, and intraoperative enteroscopy were the diagnostic tools for investigating small bowel disease [9]. Since the development of CE and DAE, the diagnosis of small bowel lesions has been revolutionized. Concurrently, the reported incidence of small bowel tumors is increasing [39]. As CE has a higher diagnostic yield than radiologic examinations, CE is regarded as the first-line examination for the evaluation of small bowel lesions [38,40,41]. CE is noninvasive, does not require sedation, and can visualize the entire small bowel [10]. Despite its noninvasiveness, CE has limitations: it cannot be used to perform tissue sampling or therapeutic interventions [10,42]. To obtain tissues for the diagnosis of small bowel lesions or to perform therapeutic endoscopy, such as polypectomy, DAE is needed. CT or MR enterography is ineffective in the detection of angioectasia or superficial inflammation, but is effective in detecting malignant small bowel tumors. If a malignant small bowel tumor is suspected, CT or MR enterography may be considered first [7]. Evidence supporting the routine use of CE is insufficient in asymptomatic patients with IDA following negative bidirectional endoscopy results [7]. Patients with IDA and negative bidirectional endoscopy had favorable outcomes without further investigation, particularly if the anemia resolved after treatment [43].

## 5. Diagnostic Yield of Capsule Endoscopy in Patients with Iron Deficiency Anemia

**Figure 2:**
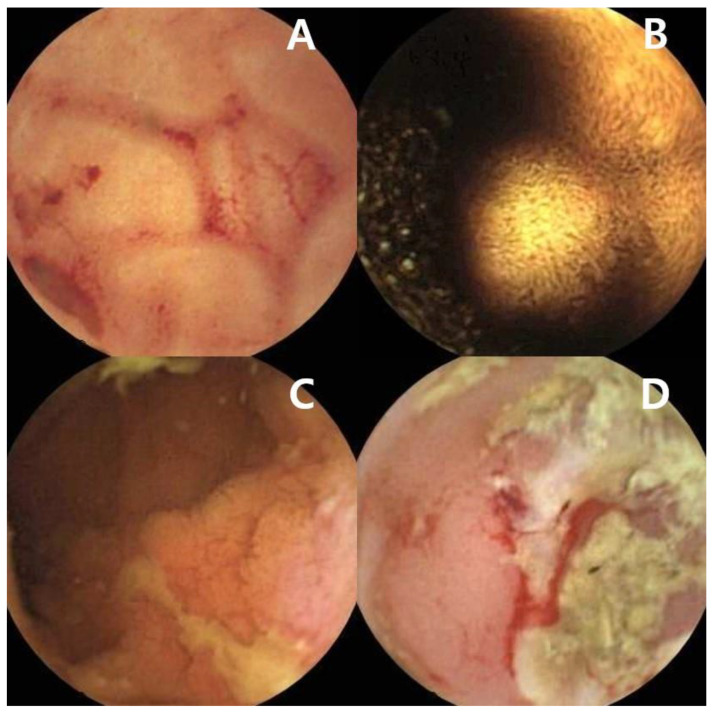
Small intestinal images captured by capsule endoscopy **(A**) Angioectasias of the jejunum. (**B**) Gastrointestinal stromal tumor of the jejunum. (**C**) Longitudinal ulcer of the ileum in a patient with Crohn’s disease. (**D**) Ileal ulcer with spontaneous bleeding in a patient with NSAID-induced enteropathy.

Despite standard bidirectional endoscopy, 30% of IDA patients are not definitively diagnosed [44]. When the standard examination results are negative, the small bowel may be considered the source of bleeding [5]. In the small bowel of IDA patients, lesions such as angioectasias, tumors, ulcers, or inflammatory lesions may be detected (Figure 2A–D) [45,46]. In a recent study by Olano et al., positive findings were present in 50% of patients with IDA; the most frequent finding was angiodysplasia [46]. According to a meta-analysis of CE in IDA patients, the pooled diagnostic yield was 66.6% when studies exclusively focused on patients with IDA were pooled [45]. However, in a recent guideline, the consensus group mentioned that the diagnostic yield of CE in unselected IDA patients is unlikely to change the long-term outcomes of patients with IDA, and CE can be considered for selected patients with more severe IDA (requiring transfusion, hemoglobin level < 10 g/dL), or recurrent or persistent IDA despite iron replacement) [47]. For patients with risk factors for small bowel malignancy, investigation of the small bowel should be considered [15,48]. Patients with FAP have an increased risk of duodenal adenomas and duodenal cancer; up to 90% of these patients have duodenal adenomas. The cumulative lifetime risk of duodenal cancer is 4–10% [15]. Duodenal polyposis needs to be assessed using upper GI endoscopy in patients with FAP. The surveillance interval is based on the Spigelman score [15,48,49]. Duodenal surveillance improved the prognosis of patients with FAP [50]. Patients with PJS have an increased risk of small bowel cancer (relative risk of 520; 95% confidence limits: 220, 1306) [51]; therefore, small bowel surveillance using CE or MRI is recommended every 1–3 years. For polyps larger than 15–20 mm in patients with PJS, elective polypectomy using DAE is recommended [15]. Some studies reported that although the diagnostic yield of small bowel lesions is significantly higher in the elderly [52], CE is also useful for the diagnosis of small bowel malignant lesions in younger IDA patients [53,54,55]. In a recent study of 220 patients with IDA (aged < 50 years) and negative bidirectional endoscopies, 32% of the patients had significant small bowel pathology, and 3.6% (*N* = 8) of the patients had a small bowel malignancy (four adenocarcinomas, three GISTs, and one lymphoma) [55]. Sidhu et al. concurred that the diagnostic yield of the younger group is lower than the diagnostic yield of the elderly, but significant lesions such as small bowel tumors are detected at younger ages, thus suggesting the usefulness of CE in the younger group [53]. The findings from Koulaouzidis et al.’s study showed that small bowel lesions of the elderly were mostly vascular lesions, such as angioectasia, but 25% of patients aged < 40 years had a sinister diagnosis of the small bowel including malignancy or Crohn’s disease [54]. In the same study, 10% of patients aged < 40 years were diagnosed with lymphoma. In a study by Johnston et al., small bowel malignancies were confirmed in seven patients (two adenocarcinomas, two GISTs, two lymphomas, and one jejunal metastasis from a lung sarcoma). The median age of the patients diagnosed with small bowel malignancies was 50 years; younger patients referred for IDA were more likely to have small bowel malignancies [56]. Considering the increased risk of complications in the elderly, it is important to determine which potential benefits might be expected from these investigations and discuss the possible therapeutic plan [37]. In a previous study of IDA patients older than 80 years, omission of additional diagnostic workup seemed appropriate in the presence of significant comorbidities and limited life expectancy [57]. Elderly patients with unexplained IDA after bidirectional endoscopic exam had favorable outcomes [58]. Girelli et al. showed that performing CE in patients older than 80 years was troublesome, and a considerable proportion of the patients experienced CE failure [59]. Therefore, CE should only be performed in selected elderly patients [37].

Importantly, although CE can be used to visualize the entire small bowel mucosa, a considerable portion of small bowel lesions, including small bowel mass, may be missed, especially in the proximal small bowel, such as the duodenum or proximal jejunum [60,61,62]. Thus, alternative diagnostic modalities, such as DAE, CT, or MR enterography, must be considered when suspicious clinical symptoms are present.

Many studies have reported on the frequency of small bowel malignancies in patients who underwent CE after negative bidirectional endoscopy for IDA (Table 1) [46,53,54,55,56,63,64,65,66,67,68,69,70,71,72,73,74,75,76]. In a study by Milano et al., 8.9% of patients with IDA were diagnosed with neoplastic disease of the small bowel. In the same study, CE missed an ileal GIST that was recognized by CT enterography, and CE recognized a small bowel metastasis from a malignant melanoma that was missed on CT enterography [63]. In a study of 51 patients with unexplained IDA, 2 patients (3.9%) were diagnosed with small bowel tumors: jejunal metastasis from a malignant melanoma and jejunal adenocarcinoma [75]. In a recent study by Kunihara et al., 8 (2.2%) of the 357 patients with unexplained IDA were diagnosed with small bowel malignancies (two adenocarcinomas, two GISTs, two lymphomas, and two small bowel metastatic cancers from hepatocellular carcinoma) [69]. According to a recent systematic review, in IDA patients after negative bidirectional endoscopy results, the pooled frequency of small bowel malignancy was 1.25% [7]. In that review, because of the inclusion of patients with symptoms and patients referred to tertiary centers for CE, the risk of selection bias was high [7]. That review concluded that in asymptomatic IDA patients with negative bidirectional endoscopy results, evidence supporting the routine use of CE was insufficient, and CE can be considered second-line approach after iron replacement therapy. However, in selected patients with refractory IDA, such as those requiring blood transfusion or with recurrent IDA or those receiving anticoagulation or antiplatelet therapy, CE can be considered first [7].

## 6. Complications of Capsule Endoscopy

CE is simple, noninvasive, and widely used. However, adverse events can occur. The main adverse event is capsule retention. Other adverse events, such as aspiration, swallowing failure, and bowel perforation, are rare and occur infrequently [77,78,79,80]. In a recent systematic review, capsule retention was found to occur in 2% of patients evaluated for small bowel bleeding, 4% of suspected IBD patients, and 8% of established IBD patients [78]. These capsule retention rates decreased by half in studies that used a patency capsule or CT enterography before performing CE [78].

Capsule retention is generally asymptomatic, and the capsule can remain in the small bowel for months or longer without symptoms [81]. Capsule retention persisting for years without symptoms has been reported [82,83]. Thus, if malignancy is not suspected, conservative management is justified [81]. In 2–3% of patients with capsule retention, obstructive symptoms may develop [78,80]. In patients with suspected Crohn’s disease, targeted medical treatment such as corticosteroids may promote capsule passage in up to 30% of patients with capsule retention [84,85]. When needed, capsule retrieval using DAE can be performed endoscopically. This has proven successful in more than 90% of capsule retention cases [86,87]. If a small bowel malignancy is suspected, surgical capsule retrieval with surgical resection is the first option [81]. The rate of capsule retention has decreased over the last 20 years [77].

Observational studies have shown that CE does not interfere with the function of pacemakers (PMs), implantable cardioverter defibrillators (ICD), or left ventricular assist devices (LVADs). Thus, CE can be safely performed in patients with implantable electromedical devices [88,89]. A recent clinical practice guideline stated that CE can be performed without special precautions in patients with PMs [47]. However, although PM and ICD do not interfere with CE, interference with the acquisition of capsule images by LVAD was reported. Thus, the capsule leads need to be separated from the implantable electromedical devices to prevent interference with the acquisition of capsule images [88].

Capsule aspiration is a rare adverse event [90,91] but can cause serious problems [92]. A fatal intracerebral hemorrhage, presumed to be related to rupture of a cerebral aneurysm that resulted from an increase in intracranial pressure due to coughing or the endoscopic capsule delivery into the duodenum, occurred in a patient with capsule aspiration [92]. In a recent comprehensive review, the aspiration rate of CE was reported to be 0.1% [93]. Of CE aspirations, 95% occurred in the elderly and 87% had significant comorbidities. Of the patients, 60% had CE aspiration symptoms, the most common of which were a cough, dyspnea, and foreign body sensation. In 77% of the patients, CE aspiration symptoms developed immediately after the capsule was ingested; however, for the remaining patients, symptoms were not apparent for hours or even days after capsule ingestion. None of the patients developed respiratory failure or significant desaturation due to capsule aspiration [93]. Coughing can stop even if the capsule device is still located within the bronchus [94]. According to a recent case report, the capsule endoscope remained in the bronchial tree for 110 days without serious problems [95]. As there have been reports that the aspirated capsule remaining [96,97], all staff involved in this procedure should be vigilant to the potential risk of tracheal aspiration of the capsule irrespective of coughing. The use of a real-time viewer may aid in the early detection and management of capsule aspiration. For patients with suspected or confirmed CE aspiration, the patient should be leaned forward and gently hit in their interscapular area to help them cough; if this fails, the next step would be to seek help from a pulmonologist who can perform a bronchoscopy [93]. Before performing CE, the identification of patients at risk of CE aspiration is required [79]. Meticulous history taking and a swallowing function test before performing CE is needed to prevent aspiration, especially in elderly patients with silent swallowing disorders or a history of cerebral stroke. In patients with an established diagnosis of swallowing disorders, endoscopic capsule delivery into the duodenum, which is safe and feasible, should be considered [98,99]. Endoscopic capsule delivery can be achieved using an overtube [100] or a special device [98].

## 7. Conclusions

Small bowel malignancies are rare and usually asymptomatic or with nonspecific symptoms. Therefore, small bowel tumors are difficult to diagnose. Small bowel tumors are serious and must not be overlooked. In IDA patients with negative bidirectional endoscopy results, CE can be considered for small bowel evaluation in selected patients with recurrent or persistent IDA. The frequency of small bowel malignancies is low in patients undergoing CE for IDA, but the usefulness of CE for the diagnosis of small bowel malignancies in younger age groups with IDA has been reported. Investigation of the small bowel should be considered in patients with risk factors for small bowel malignancy. More attention needs to be paid to the risk of capsule retention or aspiration, and efforts should be made to prevent such adverse events through meticulous history taking and endoscopic capsule delivery, as necessary.

## Figures and Tables

**Table 1:** Studies on the frequency of small bowel malignancies in patients who underwent capsule endoscopy for iron deficiency anemia.

Reference	Year	Total Patients with IDA	Small Bowel Malignancy (*N*)	Small Bowel Malignancy (%)
Romeo et al. [66]	2021	25	0	0
Singeap et al. [67]	2020	76	2	2.63
Stone et al. [68]	2020	620	2	0.32
Kunihara et al. [69]	2018	357	8	2.24
Olano et al. [46]	2018	118	4	3.39
Sealock et al. [70]	2018	75	0	0
Johnston et al. [56]	2017	805	5	0.62
Yung et al. [55]	2017	220	8	3.64
Sidhu et al. [53]	2015	971	16	1.65
Holleran et al. [71]	2013	64	0	0
Koulaouzidis et al. [54]	2012	221	2	0.90
Tong et al. [72]	2012	97	0	0
Milano et al. [63]	2011	45	4	8.89
Laine et al. [73]	2010	40	0	0
Riccioni et al. [64]	2010	138	8	5.80
Kim et al. [74]	2009	25	0	0
Muhammad et al. [65]	2009	231	0	0
Apostolopoulos et al. [75]	2006	51	2	3.92
Van Tuyl et al. [76]	2006	150	4	2.67

IDA, iron deficiency anemia.

## Data Availability

Not applicable.
